# Stable Isotope Characterization of Meat Products Through the Feed–Animal–Product Chain

**DOI:** 10.3390/molecules31193492

**Published:** 2026-10-01

**Authors:** Andrey B. Lisitsyn, Dmitriy A. Sviridov, Irina M. Chernukha, Lev A. Oganesyants, Alexandr L. Panasyuk, Mikhail Y. Ganin, Alexandr A. Il’in

**Affiliations:** 1 V.M. Gorbatov Federal Research Center for Food Systems, 109316 Moscow, Russia; info@fncps.ru (A.B.L.); i.chernuha@fncps.ru (I.M.C.); 2 All-Russian Scientific Research Institute of Brewing, Beverage and Wine Industry—Branch of V.M. Gorbatov Federal Research Center for Food Systems, 119021 Moscow, Russia; vniipbivp@fncps.ru (L.A.O.); alpanasyuk@mail.ru (A.L.P.); m-ganin@yandex.ru (M.Y.G.); tetpapx@bk.ru (A.A.I.)

**Keywords:** isotope ratio mass spectrometry, meat, pork, quail, sausages, identification, traceability, isotopic signature

## Abstract

Meat and meat products are among the most widely consumed foods worldwide. This places particular responsibility on manufacturers to ensure the quality and safety of these products. Due to their relatively high market value, meat products are susceptible to adulteration, highlighting the need for reliable methods of food authenticity assessment. Isotope ratio mass spectrometry (IRMS) method is one of the modern and most reliable analytical tools for providing information about the nature of the origin of components in food. A comprehensive analysis of isotope ratios was performed across the meat production chain, from animal feed and raw materials to finished products. Significant differences in δ^13^C and δ^15^N values were identified between pork samples collected at the two investigated sampling sites. It is shown that the isotope ratio values measured in different quail tissues and waste products ranged from −25.73‰ to −23.42‰ for δ^13^C and from 3.12‰ to 5.98‰ for δ^15^N, and are comparable with those in feeds. The δ^18^O values in meat juice ranged from −6.99‰ to −5.92‰ and were associated with the isotopic composition of local drinking water. No substantial changes in the δ^13^C and δ^15^N values of quail meat were observed following dietary supplementation under the investigated production conditions. The isotopic characteristics of individual ingredients and their potential contributions to the isotopic signature of quail meat sausages were investigated. Monitoring isotope ratios throughout the feed–animal–product chain may serve as a complementary approach for meat product identification and raw material traceability.

## 1. Introduction

In the past, food production was closely tied to a specific geographical region. Products were made of local raw materials using traditional technologies and recipes and sold in the local market. Such products were a part of the culture and everyday life of the people. In today’s globalized food market the food industry faces a crisis of trust. Consumers want to be confident in the safety, quality, and environmental friendliness of purchased products. Meat and meat products are among the main commodities in the consumer basket of a broad segment of consumers. Due to the high cost and economic attractiveness of substitution with cheaper analogs, the problem of falsification of meat products is quite acute in many countries of the world [1], and the development of identification methods is one of the priority directions in the field of food authenticity assessment. The main methods for detecting meat product falsification include the application of chromatographic analysis methods (high-performance liquid chromatography, gas chromatography) or gas chromatography-mass spectrometry. Also, visual and organoleptic assessment, DNA analysis, histological studies, spectrophotometric, and other methods are frequently used in the identification of meat and its processed products. However, in a number of cases, these methods may not be sufficient for a reliable determination of product authenticity. The most common method that provides information about the nature and geographical origin of raw materials and the products manufactured from them is isotope ratio mass spectrometry. Methods for determining the stable isotope ratios of bioelements have become widespread globally and are used to identify raw materials and finished products in the wine, dairy, and meat industries, as well as to identify honey, vegetable oils, and other products [2,3,4].

The stable isotope composition of light elements, including carbon, nitrogen, sulfur, oxygen, and hydrogen, is influenced by the botanical origin of agricultural products and environmental conditions, forming a characteristic isotopic signature. As animals consume feed and water, their tissues incorporate elements with isotopic compositions reflecting these dietary and environmental sources. The structural elements of new muscle fibers and other body tissues and water are characterized by a specific ratio of bioelement isotopes. Stable isotopes of carbon (12C and 13C), nitrogen (14N and 15N), and hydrogen (1H and 2H) do not undergo radioactive decay and maintain their ratios in biological tissues throughout the shelf life of the product [5,6,7,8].

Carbon isotope ratio 13C/12C in meat products directly depends on the type of photosynthesis of the plants that make up the animal’s diet. There are C3- and C4-pathways for atmospheric carbon dioxide fixation and CAM metabolism. The values of the carbon isotopic composition of structural components of animal tissues can provide important information about its diet before slaughter. However, the values of stable carbon isotope ratios make it possible to obtain a ‘complex’ characteristic of the diet for the period preceding slaughter. Throughout the animal’s life, its diet may change. In muscle tissue analysis, short-term dietary changes may go unnoticed. In this case, an effective approach to establishing changes in the animal’s diet involves using additional tissue samples, such as hair and hooves, which record dietary changes over time. These tissues are metabolically inert and remain unchanged, retaining the isotope ratio throughout their growth period.

Animals also receive nitrogen-containing and sulfur-containing compounds from food. Consequently, the ratios of 15N/14N and 34S/32S isotopes in the structural components of animal tissues are closely linked to their diet. Sulfates are absorbed by plant roots from the soil and are then transported to chloroplasts, where sulfur is reduced to sulfide through a series of enzymatic reactions. In animals, the assimilation of sulfur occurs through feeding; it is included in amino acids, such as cysteine and methionine, which are used to build proteins and other compounds. Nitrogen-containing compounds in agriculture are represented by compounds of biogenic and synthetic origin. This is due to the fact that soil nitrogen deficiency is felt particularly acutely. Nitrogenous fertilizers are one of the most widespread and highly demanded types of fertilizers. The most common nitrogen fertilizers include nitrate fertilizers (e.g., potassium, sodium, calcium and ammonium nitrate), urea, ammonium sulfate and ammonium chloride. At the same time, the use of nitrogen-containing synthetic fertilizers alters the isotopic composition of nitrogen in the soil. Synthetic nitrogen fertilizers exhibit characteristic δ^15^N values, which may influence the nitrogen isotopic composition of plants grown using these fertilizers. Accordingly, when using such plants as animal feed, the δ^15^N parameter will act as an isotopic label in the components of the meat.

**Determination of the geographical origin of meat.**

Products with a controlled origin are subject to stricter requirements, which ensures their high quality and uniqueness. Nevertheless, there is a risk of falsification of such products through the substitution of finished goods or raw materials with analogs from other regions. The problem of food identification is becoming increasingly urgent nowadays. This is related to ensuring food safety and quality requirements, as well as combating falsification and counterfeit goods. In this regard, studies aimed at confirming the geographical origin of food products are widely used to protect producers from unfair competition. With a stable approach to ensuring the animal feed base, the parameters 13C/12C, 15N/14N can serve as authenticity criteria for identification of products from specific farms. Isotopic characteristics of oxygen 18O/16O and hydrogen 2H/1H are dependent on fractionation processes during the formation of values of the corresponding characteristics in rainwater and groundwaters. All vegetation feeds on isotopically similar natural waters within the same geographic zone. Thus, the isotopic characteristics of plant structural components are largely determined by the unique geoclimatic factors of an individual region. It is worth noting that the oxygen and hydrogen isotope ratios in the structural components of animal tissues may depend on the proportions of fresh and dry feeds in the diet, and may also change during metabolic processes, such as sweating. In studies aimed at establishing the geographical origin of cattle and meat products, the analysis of the stable isotope ratios of carbon, hydrogen, and nitrogen is most frequently used. As described above, elements possessing an isotopic composition specific to a given region accumulate in the tissues of plants and, consequently, of the animals that consume them as feed [7,8,9,10,11,12]. However, establishing the authenticity of the geographical origin of meat products using IRMS can be complicated by a number of factors. For example, during their lifetime, agricultural animals can be raised on different farms, and they may consume feeds of various origins. The isotopic composition of bioelements in animal tissues can also experience seasonal fluctuations [13]. The feasibility of studying the isotope ratios of light elements to establish the geographical origin of beef from Japan, Australia, the United States, and European countries has also been confirmed by several studies [13,14,15,16,17,18,19].

Thus, an isotopic signature can provide important information about the animal’s diet, the method of growing forage plants, and the climatic conditions of the region of a specific farm. This method is often indispensable in cases where the physicochemical properties of a counterfeit turn out to be identical to the original product, as well as for traceability in global trade for veterinary and phytosanitary control. Unlike other studies focused exclusively on meat identification, this work evaluates the possibility of tracing isotopic ratios at all stages of the production chain—from feeds and feed additives to processed meat products.

## 2. Results and Discussion

### 2.1. Comparative Study of Isotopic Ratios of Elements in the Composition of Pork from Different Regions of Russia

The stable isotope compositions of carbon, nitrogen, oxygen, and hydrogen were analyzed in animal and plant samples collected at two sampling sites located in Belgorod Oblast and Krasnoyarsk Krai. Pork samples were obtained from both sites. In addition, samples of bone tissue, cartilage from ribs, hair, as well as a sample of compound feed, which constituted a significant proportion of the pigs’ diet, were taken from the Belgorod region. Oats and bran samples, which also constituted a significant share of the animals’ diet, were additionally selected from the Krasnoyarsk Krai. The results are presented in Table 1.

As can be seen from the table, the values of the isotopic characteristics of carbon in samples of meat, bone tissue, and cartilage of pigs selected in the Belgorod region are in a fairly narrow range from −23.10‰ to −21.07‰. These values primarily depend on the animals’ diet. At the same time, a slight fractionation of carbon isotopes in various animal tissues is observed, which is associated with the specifics of biochemical processes. Thus, the animal’s meat is characterized by a higher content of the ‘light’ 12C isotope in its structure compared to cartilage and hair samples. Meanwhile, the value of the isotopic characteristics of total carbon of the compound feed (#7), used as food for the animals, was −20.92‰. Such a value is typical for products derived from a mixture of C3 raw materials (grain, mixed grass, sunflower, soy, etc.) and C4 type plants (corn). Also, the isotopic characteristics of total carbon in this case are influenced by the characteristics of added supplements and premixes. The values of the carbon isotopic characteristics of meat samples taken in the Krasnoyarsk Krai are also in a narrow range from −26.62‰ to −26.17‰. The bulk δ^13^C value of oats and bran used to feed the animals (#11, #12) was −29.52 and −28.48‰, respectively. Such values are typical for C3 plants and their processed products. Statistical data analysis revealed significant differences between the δ^13^C isotope ratio data obtained for the two samples of pig meat (Krasnoyarsk Krai and Belgorod region). The adjusted *p*-value was 0.0057 (N = 6).

The values of nitrogen isotopic characteristics obtained for meat samples taken in Gubkin, Belgorod region, are in the range from 3.75‰ to 3.95‰. Moreover, the isotopic characteristics of nitrogen in bone tissue, cartilage, and hair have higher values compared to meat samples. Such differences may be related to the uneven distribution of nitrogen isotopes in the animal’s body, which is consistent with literature data [20,21]. The δ^15^N value for the compound feed sample (#7) is characteristic of a plant product produced using plants cultivated with synthetic nitrogen-containing fertilizers. It was previously established that industrial fertilizers are characterized by δ^15^N values close to zero (0‰), which corresponds to the isotopic characteristics of the nitrogen used for their production. Fertilizers of natural origin, conversely, have values above zero in the positive region (+1‰ to +37‰) [22]. According to Bateman et al., the content of the δ^15^N nitrogen isotope in carrots grown with ammonium nitrate is 3–4‰ [23], which is lower than when grown using bird droppings. For oat and bran samples (#11, #12), the value of this indicator was 2.59‰ and 5.81‰, respectively. Such values are also characteristic of plants cultivated using synthetic nitrogenous fertilizers. It is worth noting that in the studied samples from both regions, the bulk δ^15^N values obtained for feeds are lower than the corresponding values obtained for various tissues of animals raised on these feeds. This may be due to the presence of other additives in the animals’ diet, as well as the fractionation of isotopes during metabolism [13]. Statistically significant differences were also found between the δ^15^N isotopic ratio data obtained for meat samples from the considered regions. The adjusted *p*-value was 0.0008 (N = 6). As with the δ^13^C data, these differences are driven by different approaches to animal husbandry.

In forming the values of the isotopic characteristics of oxygen and hydrogen of the structural components of animal tissues, a major role is played by the distribution of stable isotopes in the water consumed by the animals. The isotopic characteristics of oxygen and hydrogen in water influence the isotopic composition of the structural components of animal tissues because water is the main source of oxygen and hydrogen for living organisms. In the process of metabolism, animals use water to synthesize organic substances that make up their tissues. This involves isotope fractionation. The degree of fractionation depends on many factors, such as the species of the animal, tissue type, environmental conditions, etc. Therefore, the isotopic characteristics of tissues of different animals may vary to some extent. However, no statistically significant differences were found between the δ^18^O and δD data obtained for meat from different regions. The adjusted *p*-value was 0.4 for both characteristics (N = 6 for each tested characteristic). It should be noted that the values of the isotopic characteristics of total oxygen and hydrogen of pig hair differ from the corresponding values obtained for meat samples towards ‘lighter’ δ^18^O values and ‘heavier’ δD values. Such a distribution of values is most likely related to the characteristics of isotope fractionation during the animal’s life processes. Thus, the δ^13^C and δ^15^N parameters of freeze-dried meat are of the greatest interest in terms of ensuring meat traceability, as well as confirming its geographic origin.

### 2.2. Tracing the Isotopic Signature from Feed to Final Product

#### 2.2.1. Study of δ^13^C and δ^15^N Isotopic Ratios of Feed and Additives

To prevent various diseases and maintain the functioning of the animal’s physiological systems, various biologically active additives are frequently used in enterprises. Therefore, in addition to studying the features of the distribution of isotopic characteristics of total carbon and nitrogen in the main feed, it is necessary to study these indicators in the additives used at the enterprise, as well as to assess the contribution of the values obtained for additives to the values obtained for the finished feed mixture.

The isotopic characteristics of total carbon and nitrogen of main feed samples, biologically active additives used for feeding—DAFS-25 (an additive based on diacetophenonyl selenide) and SelMet (an additive based on selenomethionine), as well as in prepared mixtures of the main feed and these additives, were studied. The results are presented in Table 2.

It was found that when additives are introduced into feed, the δ^13^C parameter of the obtained mixture changes slightly. Most likely, this is due to the small dosage of the preparation itself and the low concentration of carbon-containing compounds in its composition. Nevertheless, some spread of the obtained values is observed. This is typical for a multicomponent product. Despite homogenization, the complexity of the analyzed product’s composition can affect the convergence of results. As for the δ^15^N indicator, for feed samples the obtained values are in a fairly narrow range from 4.41‰ to 4.76‰. At the same time, nitrogen was not detected in dietary supplement samples #4 and #5. In sample #4, the main carrier is calcium phosphate. The content of L-selenomethionine in the preparation is less than 0.01%, in the molecule of which nitrogen is represented by one NH2 amino group. Such a concentration is insufficient for analysis. Accordingly, the effect of nitrogen in the composition of the SelMet preparation on the values of the isotopic ratios of total nitrogen in the feed mixture is insignificant. In the DAFS-25 preparation, nitrogen-containing substances are not declared at all. This is indirectly confirmed by these studies. As expected, when conducting isotopic analysis, nitrogen was also not detected in its composition. Thus, from the results of the conducted study, it can be concluded that the values of the isotopic characteristics of carbon and nitrogen in the composition of the initial feed make the main contribution to the values of the resulting mixture. Accordingly, dietary supplementation did not substantially affect the δ^13^C and δ^15^N values.

#### 2.2.2. Traceability of the Main Raw Material in a Meat Product by Isotopic Ratios δ^13^C and δ^15^N

The possibility of tracing isotopic ratios in the feed–animal–product system was studied on the example of quail meat production and its processed products. At this stage of the work, the main task was to establish the ranges of typical values of carbon and nitrogen isotope ratios in the composition of structural components of quail meat, taking into account the specifics of its production. At the same time, it is worth considering the possible change in the studied parameters of meat structural components over time. The use of biologically active additives can lead to isotopically uneven assimilation of mixed feed, which over time can cause a shift in isotopic characteristics. The δ^13^C and δ^15^N values of quail meat were compared between groups receiving the main diet or diets supplemented with different feed additives. Samples were collected after 15 and 30 days of feeding. The results are presented in Table 3.

From the results obtained, it is evident that the measured values for the groups of quails sampled at different stages of fattening are in similar ranges. Statistical data processing was performed using Student’s *t*-test. When comparing samples after 15 and 30 days of fattening by the δ^13^C parameter, the adjusted *p*-value was 0.08 (N = 10). When comparing samples after 15 and 30 days of fattening by the δ^15^N parameter, the adjusted *p*-value was 0.85 (N = 10). Thus, no statistically significant differences were observed after correction for multiple testing. The obtained values of both samples are in a fairly narrow range and fit into the established range for feeds. Also within a fairly narrow range are the values obtained for groups of quails whose diets included biologically active additives. In this regard, all the obtained bulk δ^13^C and δ^15^N values of meat are considered typical for this production. Thus, the ranges of values for the δ^13^C parameter were from −24.56‰ to −24.14‰, and for the δ^15^N parameter from 4.21‰ to 5.50‰. Meanwhile, the values of isotopic characteristics obtained for meat from the Krasnoyarsk Krai (Table 1), for the δ^13^C parameter were in the range from −26.62‰ to −26.17‰, and for the δ^15^N parameter from 7.51‰ to 7.91‰. Such differences in value ranges are primarily due to the use of different feeds at specific enterprises. Thus, the δ^13^C and δ^15^N parameters are important parameters forming the ‘isotopic signature’ of meat products.

The indicators of carbon and nitrogen isotope ratios of structural components in meat identification have attracted considerable scientific interest among researchers from various countries with developed animal husbandry. Thus, in different countries of the world, δ^13^C and δ^15^N isotopic ratios in meat are in different numerical ranges, which is due to cultural and geographical features in the approach to forming the feed base within local livestock farming [9,24,25,26,27,28]. Thus, in the work of Kim K. S. et al. [25], it is shown that the δ^13^C values of meat from various countries are ranked in the following sequence: America (−15.55 ± 1.01‰) > Korea (−19.62 ± 0.89‰) > Canada (−22.87 ± 0.92‰) > Europe (−24.79 ± 1.35‰). In the work of Bahar B. et al. [26], studying Irish beef samples revealed a seasonal shift in δ^13^C values by more than 2‰ between December and June, while the δ^15^N values remained virtually unchanged (around 7‰). In this study, the isotopic characteristics of total carbon and nitrogen in various types of tissues and waste products of quails raised on a specific farm were also investigated. As established, the isotopic characteristics of elements of structural components in the composition of the animal’s meat are formed primarily based on the features of the distribution of these parameter values in the feed used. However, during the formation of animal tissues, the process of isotopic element fractionation can occur; therefore, the distribution of ‘light’ and ‘heavy’ isotopes in the structures of different animal tissues may proceed unevenly. In this regard, to obtain a complete picture of the distribution of isotopic ratios of total carbon and nitrogen, in addition to quail meat, samples of heart, liver, paws, feathers, fat, eggs, and droppings were studied. As in the case of meat, the values obtained for samples of different tissues sampled 15 and 30 days after the start of feeding were in similar ranges. Accordingly, the values obtained for each type of tissue were calculated as a single sample. The results are presented as boxplots in Figure 1 and Figure 2.

The ranges of carbon isotopic characteristics values for all studied samples were from −25.73‰ to −23.42‰. The values of isotopic characteristics obtained for the main diet are within the established range. The highest obtained values were noted for quail eggs. The lowest values were observed in quail fat. It is worth noting that the formation of muscle tissue, liver, and heart of the animal occurs as a result of similar metabolic processes. The composition of droppings is also directly related to metabolic processes. The obtained values of carbon isotopic ratios in these samples are comparable with the values obtained for feeds. At the same time, the formation of adipose tissue occurs as a result of special metabolism, which is confirmed by the obtained isotopic ratio data.

The ranges of values for the nitrogen isotopic characteristics of the studied samples ranged from 3.12‰ to 5.98‰. The established values are consistent with those obtained for feeds. At the same time, the δ^15^N data obtained for muscle tissue, heart, and liver are also in identical numerical ranges. The highest average values were recorded for quail droppings. The lowest—for feathers. During the formation of feather tissue, the values of isotopic characteristics of their structural components are fixed at the moment of their formation and do not change during life. Thus, the low values obtained may reflect the characteristics of the feeding diet prior to the start of the study. It should be noted that there is a lack of extensive studies globally aimed at studying the fractionation of stable isotopes of elements during the life activity of animals, including the formation of various types of tissues. Summarizing, we can conclude that the δ^13^C and δ^15^N isotope ratio values obtained for different tissues are in fairly narrow ranges. The differences identified may be due to isotopic fractionation processes during the formation of tissues and waste products.

In the conducted study, the values of isotopic characteristics of total carbon and nitrogen of egg white specifically were presented. This is because the carbon- and nitrogen-containing compounds in egg white are almost entirely represented by proteins. When identifying identification markers, preference is usually given to a monoproduct. However, to obtain more comprehensive data, a comparative study of the δ^13^C and δ^15^N values of egg white and egg yolk was conducted. Based on the results, differences in the δ^13^C values were found for the white and yolk of the same egg. The values obtained for egg white were −23.62 ± 0.01‰ and were close to the values obtained for dried meat (Table 3). The composition of these samples is largely represented by proteins. The composition of the egg yolk is largely represented by fats. Accordingly, the δ^13^C values obtained for the yolk (−25.27 ± 0.07‰) fall within the range established for adipose tissue. No significant differences in the δ^15^N values obtained for egg white and yolk were detected (4.33 ± 0.25‰ and 4.20 ± 0.80‰, respectively). This observation is confirmed in the current study, which found that the δ^15^N values for fat and quail meat are in similar ranges (Figure 2).

#### 2.2.3. Study of δ^18^O and δD Isotopic Ratios of Water Samples and Meat Juice

During animal rearing, in addition to feed, water is also used. Within one geographical area, groundwater is usually characterized by similar isotopic characteristics of oxygen and hydrogen. This is due to the fractionation processes of oxygen 18O/16O and hydrogen 2H/1H isotopes in water molecules occurring during the formation of both rainwater and groundwaters in a specific geographic zone. Drinking water used in animal feeding also has an ‘isotopic signature’ typical of a particular region. In this regard, studying the isotopic characteristics of local water is the starting point in analyzing the features of oxygen and hydrogen isotopic ratios of water in meat and meat products. To identify the ranges of typical values of the isotopic characteristics of oxygen and hydrogen of local water (Volgograd), water samples taken from three different local springs, samples of water used for feeding quails, and samples of rainwater were studied. All samples were taken in duplicate with a time difference of 10 days between sampling. The results are presented in Table 4.

Rainwater samples were included to assess possible isotope fractionation processes within the local water cycle and to facilitate interpretation of the oxygen isotope composition of the investigated biological samples.

The conducted studies showed that the values of the isotopic characteristics of oxygen and hydrogen of water body samples, taken with a difference of 10 days, differ insignificantly, with the exception of rainwater samples. The obtained data are consistent with previous studies, which showed that the values of oxygen and hydrogen isotopic characteristics of ground and tap waters are relatively stable. Their changes can occur smoothly over a long time due to the influence of large-scale geoclimatic processes. Thus, the range of local water values for the specific production (Volgograd) in terms of δ^18^O was from −13.05‰ to −12.03‰, and in terms of δD from −97.93‰ to −77.79‰.

In the data provided in the literature, as a rule, values of the isotopic characteristics of oxygen in water are studied, since this indicator is more stable compared to the isotopic characteristics of hydrogen. According to Franke B. M. et al. [28], the δ^18^O values of water in Central European countries range from −8‰ to −11‰, while for Brazil, this range is from −2‰ to −10‰. Thus, water from the studied region (Volgograd region) is characterized by a higher concentration of the 16O isotope compared to water from some European countries and Brazil.

It is necessary to separately consider the values obtained for rainwater sampled on different days. The isotopic characteristics values of oxygen and hydrogen obtained for rainwater differ significantly from similar values obtained for spring and drinking waters from the same region. This is associated with the specifics of the formation of the isotopic composition of rainwater. Water molecules containing lighter isotopes generally have higher vapor pressures than their heavier isotopic counterparts under comparable conditions. Thus, the vapor pressure of H_2_O^16^ molecules is higher than the vapor pressure of D_2_O^18^ molecules. In this connection, the water vapor formed during the evaporation of liquid water is enriched with 16O and 1H atoms. Upon condensation of water vapor in clouds, the reverse effect occurs; water drops become enriched with ‘heavy’ 18O and D isotopes, which leads to an even greater increase in the concentration of ‘light’ 16O and 1H isotopes in the remaining vapor. With further movement of clouds with air masses, a similar cycle occurs. Thus, the values of oxygen and hydrogen isotopic characteristics of rainwater depend on the features of the water area near which the clouds were formed, as well as on the distance they have traveled. Accordingly, at the same sampling location, rainwater collected on different days can be characterized by different values of the isotopic characteristics of elements in its composition. For instance, sample #4 from August 14 is characterized by higher values of oxygen and hydrogen isotopic ratios compared to the sample taken on August 4. It can be assumed that the sample from August 14 represents the first raindrops from clouds formed over local water bodies, while the sample from August 4 had already undergone fractionation during the movement of water vapor. Considering that purified drinking water was used when raising quails and producing meat products, we adopt the values δ^18^O—−12.07‰ and δD—−85.60‰ as typical for the given region.

The next stage of the work consisted of studying the isotopic characteristics of oxygen and hydrogen of the water directly within the composition of quail meat. The values of isotopic characteristics were measured using the GasBench II isotopic equilibration system. The obtained oxygen isotopic characteristics values for meat juice extracted from minced quail meat range from −6.99‰ to −5.92‰. The hydrogen isotopic characteristics values for these same samples are in the range from −73.20‰ to −66.36‰. The average values typical for the water component of meat from the specified region were −6.59‰ for δ^18^O and −69.99‰ for δD. The obtained spread of values is most likely related to the physiological characteristics of specific individual quails. It is shown that the water in quail tissues is characterized by a higher content of ‘heavy’ oxygen and hydrogen isotopes in comparison with drinking water. Such an observation is typical for animals and is also related to isotopic fractionation during their life processes. The obtained values have a close relationship with the features of forming isotopic element ratios within local water and depend on the geographic location of production. Thus, based on the obtained results, it can be concluded that the identified ranges are characteristic of the aqueous component of meat from quails raised in the conditions of a specific enterprise, taking into account the specifics of the isotopic composition of local water. In the data cited in the literature sources, typically only the values of the oxygen isotopic characteristics are presented. This is because hydrogen isotope analysis using the GasBench II system is labor-intensive. At the same time, δ^18^O and δ^2^H are strongly correlated as a result of isotopic fractionation [29,30]. It is worth noting that the established δ^18^O range of the aqueous component of meat juice also has some differences when compared with similar data obtained by researchers for certain European countries and Brazil [28]. At the same time, differences between the δ^18^O values of meat juice and local water δ^18^O are comparable. Such differences are most likely related to the characteristics of the isotopic composition of local water, dictated by the geoclimatic parameters of the animal rearing region, which, in turn, points to the possibility of classifying products manufactured in different geographic zones based on this parameter. The identified ranges are more applicable for the direct identification of meat. However, studying the aqueous component can serve as one of the tools in a comprehensive approach to assessing the authenticity of its processed products using the isotope mass spectrometry method.

#### 2.2.4. Study of δ^13^C and δ^15^N Isotopic Ratios in Sausages and the Formation of the Isotopic Signature

The next stage of the work included studying the isotopic characteristics of total carbon and nitrogen of structural components in the finished product. In this case, one should consider the possible influence of technological processing methods of raw materials (heating, mixing, extraction, fermentation, etc.) on the change in the stable isotope ratios of the initial ingredients. Thus, using the example of noodles, a decrease (*p* < 0.05) in δ^2^H by 3.8‰ was shown when keeping noodles for 6 min in the interval from 100 to 260 °C with an interval of 80 °C. The δ^18^O value was affected by heating above 180 °C [31]. As is known, these values take into account the features of the distribution of isotopic ratios of the indicated elements of all product components. In this connection, to evaluate the influence of added recipe components on the final values, a study of the bulk δ^13^C and δ^15^N isotopic ratios in the finished product, as well as all components entering its composition, was conducted. The results are presented in Table 5.

The results obtained for sausage samples by the δ^13^C parameter lie in a fairly narrow range from −24.59‰ to −24.41‰. This range practically coincides with the range established for quail meat. Naturally, this range occupies an intermediate position on the numerical axis relative to the obtained values for other recipe components in the composition of the sausages, since the bulk values of carbon isotopic ratios in the finished product are formed from the aggregate of the values of this parameter for the components in its composition. The δ^15^N values of sausage samples are in a wider range—from 4.70‰ to 5.75‰. The results obtained are also consistent with the carbon and nitrogen isotopic ratio data of the ingredients used in the formulation. It should be noted that for the studied meat samples, a wider range of δ^15^N values is also observed. At the same time, the spread of the obtained values for feeds is significantly smaller. This may be due to the characteristics of isotopic fractionation resulting from the life activities of specific individual quails. Nevertheless, based on the results of the study, it can be concluded that the δ^13^C and δ^15^N isotope ratio indicators are capable of providing a high level of traceability in the feed–animal–product system.

Additionally, the δ^18^O and δD values of the water included in the sausage recipe were investigated. The δ^18^O value was −12.41‰, and the δD value was −93.13‰. The obtained results are consistent with the element isotopic ratio data in the local water (Table 4). The δ^18^O and δD indicators of water within the finished product can also serve as identification criteria. In this case, conducting an isotopic ratio analysis will be difficult since the water will be in a bound state and its separation requires special sample preparation procedures. Nonetheless, studying the isotopic composition of the aqueous fraction provides additional information about the origin of raw materials and the conditions of product manufacturing, since these parameters reflect the specificities of local water resources.

At the same time, to form a complete ‘isotopic signature’ of a meat product, it is necessary to primarily consider the distribution of stable isotopes of carbon and nitrogen in the organic part of the product. Since the final isotopic ratio values result from the contribution of all recipe ingredients, predicting the isotopic signature of the final product based on the known isotopic characteristics of its components is of special scientific interest. In some studies, machine learning methods are applied to solve this problem [32,33]. However, in most cases, changes in isotopic composition are linear. In this regard, an isotopic-material balance model for multicomponent products was developed:

$$\delta^{13}C_{mix}=\frac{\sum_{i=1}^{n}\;\left(\omega_{i}\cdot C_{i}{\cdot \delta }^{13}C_{i}\right)}{\sum_{i=1}^{n}\;\left(\omega_{i}{\cdot C}_{i}\right)}$$

$$\delta^{15}N_{mix}=\frac{\sum_{i=1}^{n}\;\left(\omega_{i}{\cdot N}_{i}{\cdot \delta }^{15}N_{i}\right)}{\sum_{i=1}^{n}\;\left(\omega_{i}{\cdot N}_{i}\right)}$$

where

ωi—proportion of the component in the product;
Ci, Ni—proportion of carbon and nitrogen in the structure of the component;
δ^13^Ci, δ^15^Ni—values of carbon and nitrogen isotopic ratios in the component.

While machine learning approaches may offer improved classification performance for large datasets, the proposed mass-balance model provides a transparent and physically interpretable description of isotopic signature formation in multi-component meat products. This feature may be advantageous when investigating the influence of individual ingredients on the isotopic characteristics of the final product. The proposed model should be considered as a conceptual framework describing the formation of isotopic signatures in multi-component meat products. Further development and evaluation of the approach may include the incorporation of additional compositional information, such as carbon and nitrogen contents of individual ingredients, which were not investigated in the present study.

## 3. Materials and Methods

Pork samples were collected from Belgorod region and Krasnoyarsk Krai—two regions with different natural and climatic conditions and distinct characteristics of livestock farming practices. In Belgorod region, samples of bone tissue and hair were additionally taken. Cartilage was separated from the bone tissue samples. The samples were dried in an oven to constant weight at a temperature of 60 °C, and then homogenized. In the Volgograd region, samples of quail meat and other tissues (liver, heart, feathers, droppings, fat, paws, eggs), feed and supplements, samples of local water and liquid in the quails’ composition, finished quail products (sausages), and recipe components were taken. Quails were selected as a model system because the investigated farm operates a complete production chain, from poultry rearing and feed supplementation to the manufacture of finished meat products. Quail sausages were selected as a representative multicomponent processed meat product. The study was conducted using quails raised at a commercial poultry farm. Dietary supplementation was part of the farm’s routine feeding practices and did not involve invasive procedures or other experimental interventions requiring separate approval from an animal ethics committee.

The calculated nutritional value per 100 g of compound feed was: 270 kcal of metabolizable energy, 17.03 g of crude protein, 3.82 g of crude fat, 4.97 g of crude fiber, and 12.48 g of crude ash. Individual pigs and quails were selected from different batches taking into account the principles of representative sampling. The pressed meat juice was clarified by centrifugation on an ELMI CM 6M centrifuge (ELMI SIA, Riga, Latvia) for 10 min at 3000 rpm. Samples of pig meat, quail tissues, and sausages were dried using a Sonyan SY-012E freeze dryer (Beijing Songyuan Huaxing Technology Development Co., Ltd., Beijing, China) with subsequent homogenization using a Stegler LB-2 (China). The sampling locations are shown in Figure 3.

In the Delta V Advantage mass spectrometer configuration (Thermo Fisher Scientific, Bremen, Germany) coupled with the Flash HT EA-MS elemental analyzer, the values of isotopic characteristics of nitrogen δ^15^N_Air_, carbon δ^13^C_VPDB_, oxygen δ^18^O_VSMOW2_, and hydrogen δD_VSMOW2_ were measured. In the case of analyzing carbon and nitrogen isotope ratios, the samples were encapsulated in tin capsules and burned in a redox reactor in a helium flow (100 mL/min) with the addition of a portion of pure oxygen. Then, the N_2_ and CO_2_ gases went through the ConFlo VI interface to the ion source of the isotope mass spectrometer for isotopic analysis. The standard sample weights were 0.2–0.8 mg. For the analysis of hydrogen and oxygen isotopic ratios, similar sample preparation procedures were used. The samples were encapsulated in silver capsules. The analysis was conducted using a pyrolytic reactor. During the measurement, hydrogen and oxygen formed during the combustion of the sample were carried by the helium carrier gas. Further, this mixture passed through a glassy carbon reactor at a temperature of 1400 °C. The reactor is a ‘tube in tube’ system (a glassy carbon tube inside a ceramic tube), where the oxidation process is prevented. The δ^13^C and δ^15^N isotope ratio values for meat were calculated as averages for the 10 studied samples. The measurement of oxygen isotope ratios of the aqueous component in meat juice was conducted using an isotope mass spectrometer coupled with the GasBench II module. The method is based on isotopic equilibration of water molecules from the analyzed sample and gas mixture molecules. Isotopic equilibration took place in closed tubes for 20 h in a thermostat at a temperature of 24 °C.

Student’s *t*-test and Welch’s *t*-test were used to compare data between two groups (significance level was set at α = 0.05). Welch’s *t*-test was applied when the sample size was less than five observations in either group. Homogeneity of variances was assessed using Levene’s test. Multiple-comparison correction was performed using the Holm–Bonferroni method. The work was carried out in the Python 3.9.13 environment, pingouin and statsmodels libraries were used for statistical analysis.

## 4. Conclusions

The present study demonstrated the applicability of isotope ratio mass spectrometry for investigating the formation of isotopic characteristics in the feed–animal–product system. The obtained results showed that δ^13^C and δ^15^N values consistently reflect the characteristics of animal feed and can be used to trace the origin of raw materials throughout different stages of the production chain. The δ^18^O values of the aqueous component of meat were found to be associated with the isotopic composition of consumed water and site-specific environmental conditions, indicating their potential usefulness as an additional criterion for assessing the origin of meat products. The results confirm the value of a multi-isotope approach based on carbon, nitrogen, and oxygen isotope ratios for characterizing the isotopic signature of meat products. The study also demonstrated that reliable interpretation of isotopic characteristics in processed meat products requires consideration of the contribution of individual recipe components and the properties of the raw materials used. Several limitations should be acknowledged. The study was conducted on a limited number of production systems and sample types, which may restrict the broader applicability of the observed relationships. Future research should focus on expanding the geographical coverage and sample size, as well as developing reference databases of isotopic characteristics for feeds, livestock products, and processed meat products. Overall, the findings indicate that stable isotope analysis can serve as a complementary tool for the identification, authenticity assessment, and traceability of meat products within food quality control systems.

## Figures and Tables

**Figure 1:**
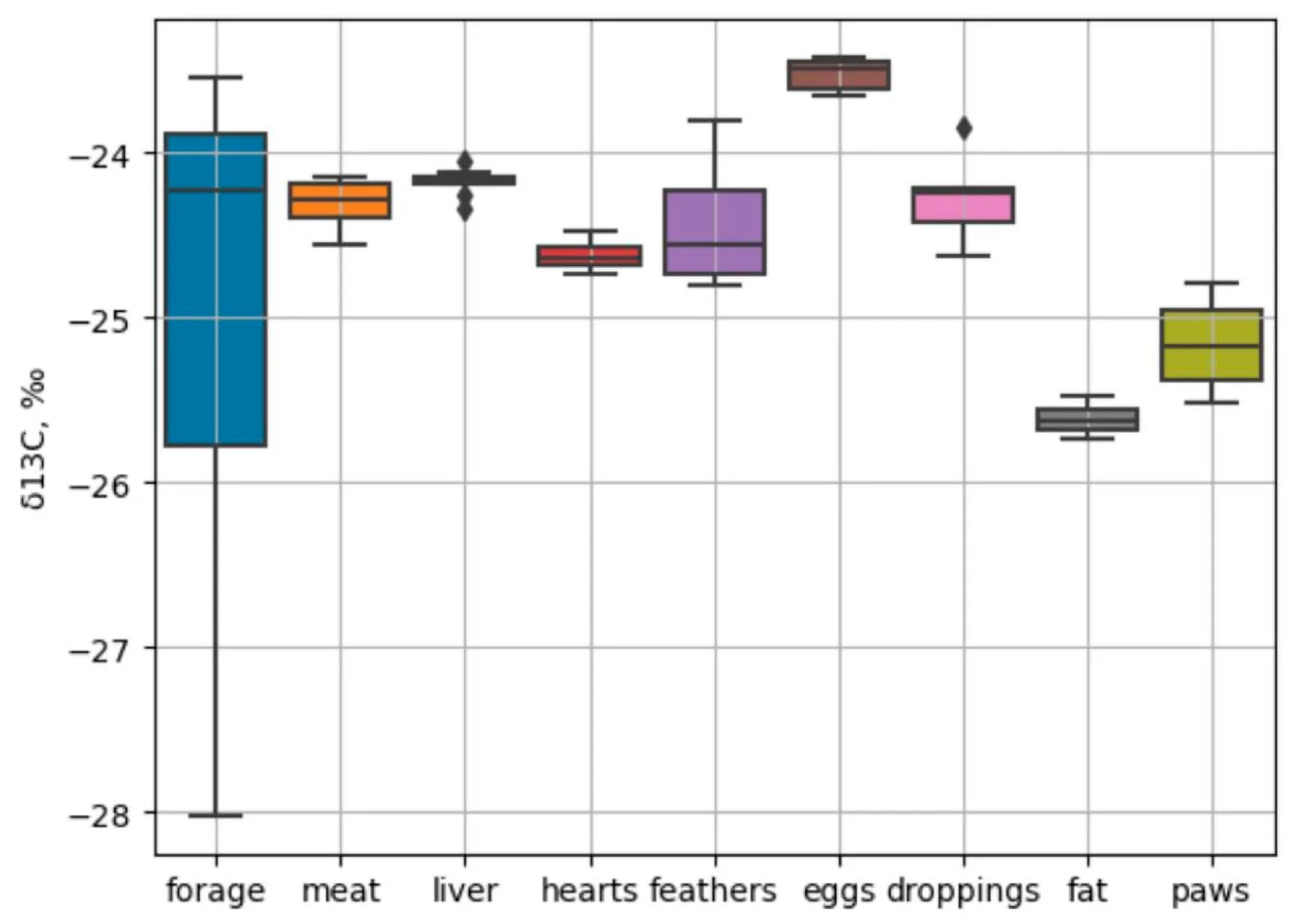
Values of isotopic characteristics of total carbon in the studied samples.

**Figure 2:**
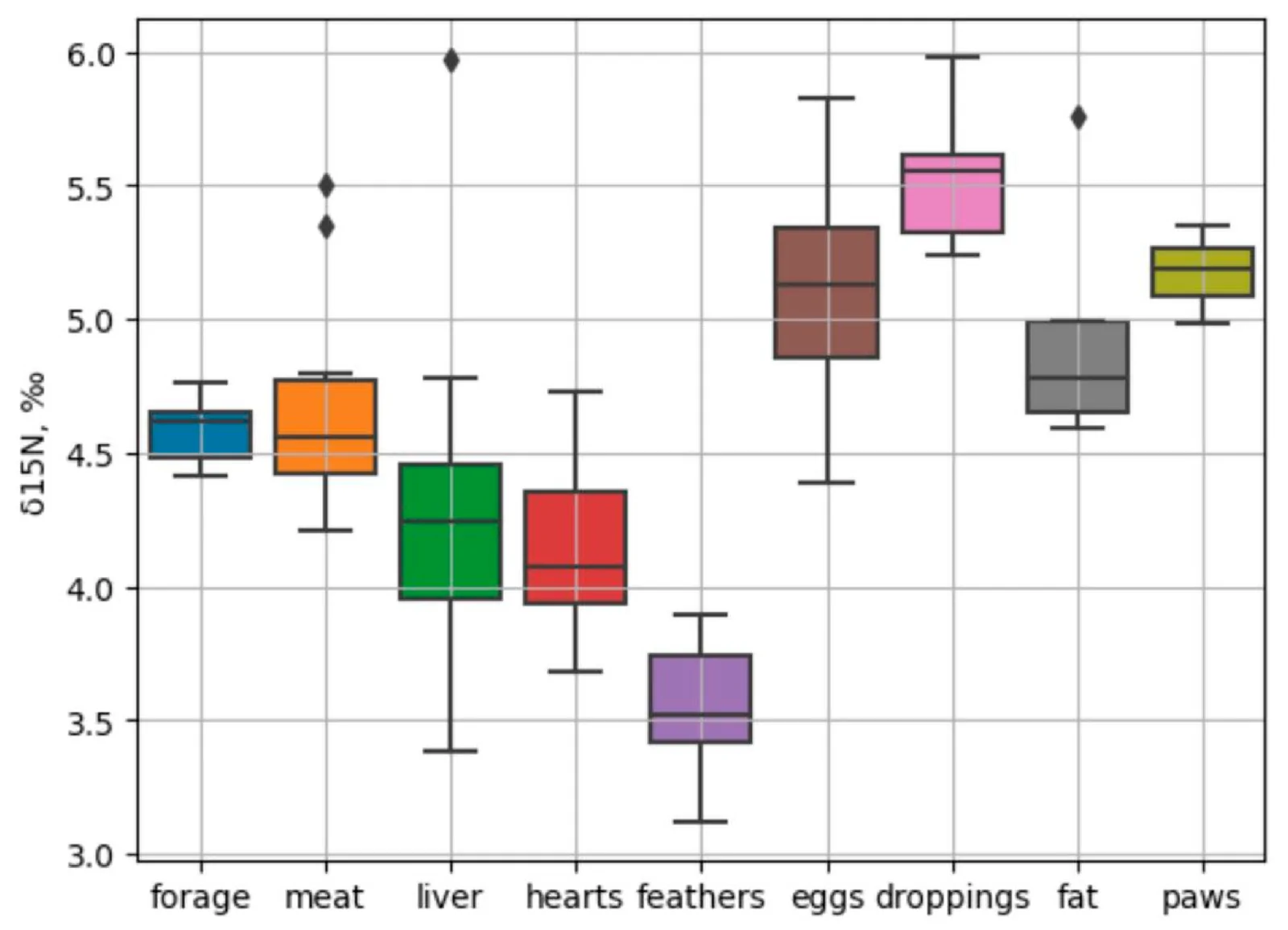
Values of isotopic characteristics of total nitrogen in the studied samples.

**Figure 3:**
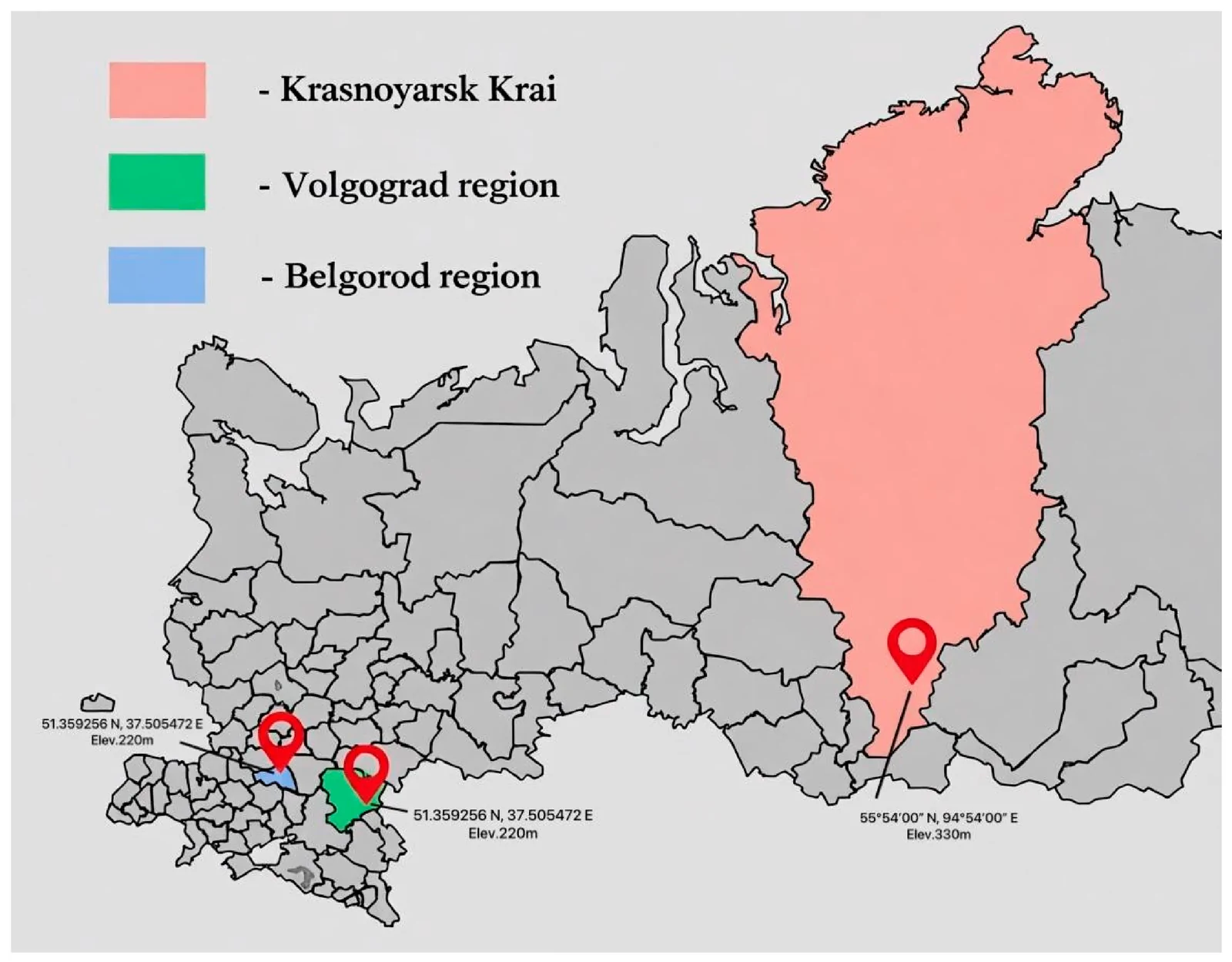
Geographical locations of sampling.

**Table 1:** Values of isotopic characteristics of total carbon, nitrogen, oxygen, and hydrogen of selected samples of animal and plant origin.

No.	Sample Name	δ^13^C, ‰	δ^15^N, ‰	δ^18^O, ‰	δD, ‰
Belgorod region					
1	Pork #1	−22.28 ± 0.05	3.86 ± 0.06	8.11 ± 0.67	−135.32 ± 1.12
2	Pork #2	−22.17 ± 0.21	3.95 ± 0.28	8.19 ± 1.03	−133.16 ± 2.00
3	Pork #3	−23.10 ± 0.14	3.75 ± 0.19	8.75 ± 1.10	−170.65 ± 5.92
4	Bone tissue, ribs	−22.75 ± 0.16	4.68 ± 0.06	8.28 ± 0.81	−155.69 ± 2.08
5	Cartilage from ribs	−21.20 ± 0.07	4.98 ± 0.13	9.19 ± 0.61	−109.83 ± 8.07
6	Pig hair	−21.07 ± 0.05	5.19 ± 0.22	5.24 ± 1.36	−106.25 ± 4.22
7	Compound feed	−20.92 ± 0.28	1.47 ± 0.40	28.26 ± 1.08	−86.94 ± 7.45
Krasnoyarsk Krai					
8	Pork #4	−26.61 ± 0.10	7.91 ± 0.04	9.75 ± 0.83	−166.04 ± 3.95
9	Pork #5	−26.43 ± 0.27	7.51 ± 0.27	9.00 ± 0.35	−174.08 ± 4.69
10	Pork #6	−26.17 ± 0.33	7.54 ± 0.14	8.35 ± 0.62	−165.37 ± 2.84
11	Oats	−29.52 ± 0.26	2.59 ± 0.13	26.62 ± 0.64	−127.97 ± 7.04
12	Bran	−28.48 ± 0.18	5.81 ± 0.41	26.34 ± 1.45	−129.41 ± 4.20

**Table 2:** Values of isotopic characteristics of total carbon and nitrogen in the composition of feeds and additives.

No.	Name	δ^13^C, ‰	δ^15^N, ‰
1	Main diet	−24.17 ± 0.14	4.48 ± 0.16
2	Main diet + DAFS-25	−25.00 ± 0.38	4.76 ± 0.14
3	Main diet + SelMet	−23.79 ± 0.66	4.58 ± 0.27
4	SelMet	−26.56 ± 0.09	Not detected
5	DAFS-25	−28.02 ± 0.02	Not detected

**Table 3:** Values of isotopic characteristics of total carbon and nitrogen in the composition of freeze-dried quail meat.

No.	Sample Name	δ^13^C, ‰	δ^15^N, ‰
Freeze-dried quail meat after 15 days of fattening			
1	Quail meat #1	−24.26 ± 0.10	4.44 ± 0.21
2	Quail meat #2	−24.14 ± 0.03	4.41 ± 0.01
3	Quail meat #3	−24.36 ± 0.08	4.64 ± 0.03
4	Quail meat #4	−24.18 ± 0.06	4.25 ± 0.20
5	Quail meat #5	−24.18 ± 0.05	5.50 ± 0.12
Freeze-dried quail meat after 30 days of fattening			
6	Quail meat #6	−24.41 ± 0.08	5.35 ± 0.12
7	Quail meat #7	−24.48 ± 0.09	4.68 ± 0.32
8	Quail meat #8	−24.32 ± 0.08	4.80 ± 0.38
9	Quail meat #9	−24.22 ± 0.03	4.47 ± 0.26
10	Quail meat #10	−24.56 ± 0.06	4.21 ± 0.23

**Table 4:** Values of isotopic characteristics of oxygen and hydrogen of the studied water samples.

No.	Sample Names	From 4 August 2025		From 14 August 2025	
		δ^18^O, ‰	δD, ‰	δ^18^O, ‰	δD, ‰
1	Spring #1	−12.90 ± 0.40	−77.79 ± 2.70	−12.72 ± 0.06	−97.93 ± 0.44
2	Spring #2	−12.87 ± 0.51	−85.79 ± 1.76	−13.05 ± 0.10	−89.30 ± 0.60
3	Spring #3	−12.16 ± 0.35	−82.72 ± 0.58	−12.16 ± 0.07	−85.78 ± 1.82
4	Rainwater	−4.57 ± 0.24	−27.31 ± 0.50	4.26 ± 0.15	11.11 ± 0.05
5	Drinking tap water	−12.03 ± 0.56	−84.45 ± 1.72	−12.12 ± 0.46	−86.03 ± 1.45

**Table 5:** Values of isotopic characteristics of total carbon and nitrogen in the composition of produced sausages and components for their production.

No.	Name	Share in Product, %	δ^13^C, ‰	δ^15^N, ‰
1	Sausages	100	−24.51 ± 0.21	5.27 ± 0.25
2	Quail meat	65.53	−24.31 ± 0.14	4.67 ± 0.44
3	Quail egg	7.31	−24.44 ± 0.03	4.27 ± 0.61
4	Milk powder	10.91	−23.77 ± 0.21	5.56 ± 0.16
5	Butter	10.91	−25.24 ± 0.19	5.71 ± 0.04
6	Onion	2.96	−26.35 ± 0.63	2.32 ± 0.63
7	Paprika	1.10	−26.42 ± 0.22	Not detected

## Data Availability

The original contributions presented in this study are included in the article. Further inquiries can be directed to the corresponding author.
